# On-Body Sensor Positions Hierarchical Classification

**DOI:** 10.3390/s18113612

**Published:** 2018-10-24

**Authors:** Vu Ngoc Thanh Sang, Shiro Yano, Toshiyuki Kondo

**Affiliations:** Department of Computer and Information Sciences, Tokyo University of Agriculture and Technology, Tokyo 184-8588, Japan; vsang@livingsys.lab.tuat.ac.jp (V.N.T.S.); syano@cc.tuat.ac.jp (S.Y.)

**Keywords:** sensor position, inertial measurement unit, feature selection, fractal dimension, hierarchical classification

## Abstract

Many motion sensor-based applications have been developed in recent years because they provide useful information about daily activities and current health status of users. However, most of these applications require knowledge of sensor positions. Therefore, this research focused on the problem of detecting sensor positions. We collected standing-still and walking sensor data at various body positions from ten subjects. The offset values were removed by subtracting the sensor data of standing-still phase from the walking data for each axis of each sensor unit. Our hierarchical classification technique is based on optimizing local classifiers. Many common features are computed, and informative features are selected for specific classifications. In this approach, local classifiers such as arm-side and hand-side discriminations yielded F1-scores of 0.99 and 1.00, correspondingly. Overall, the proposed method achieved an F1-score of 0.81 and 0.84 using accelerometers and gyroscopes, respectively. Furthermore, we also discuss contributive features and parameter tuning in this analysis.

## 1. Introduction

With the advancement of technology, there has been a growing trend towards using motion sensors in the healthcare area. Sensors such as an accelerometer and a gyroscope, which are embedded in commercial devices like inertial measurement units (IMUs) or a smartphone, provide useful contextual information [1,2]. These data play an important role in activity recognition [3,4,5,6], gait analysis [7,8,9] and passive health monitoring [10]. In recent years, the increase of smart devices using wearable sensors has led to the need of sensor position detection. For example, a smartwatch must be placed on a specific forehand in a determined orientation to function properly.

Motion sensor-based studies and applications are typically position- and orientation-dependent approaches. In many experiments, the positions and orientations of sensor are predetermined and assumed unchanged or fixed using straps when subjects perform several activities [11,12,13] because a change in the on-body sensor position can make the estimation algorithm inaccurate [14]. Many studies have proposed their works as a position-independent solution. For instance, the work [15] trained data from all relevant positions on an individual during stationary, walking, running, biking, or in motorized transport. The paper [16] presented a ready-to-use activity recognition system in both offline and online contexts. The experiment recorded data from all target positions and used orientation-independent features for classification. Equally important, the orientation-independent approach has been studied permitting the user’s freedom when using mobile devices. This approach alters between implementing orientation-independent features and transforming signal [5]. For example, the common feature in the former alternative is acceleration magnitude instead of the individual three-axis of an accelerometer [16]. In the latter alternative, the local coordinate system of the sensor is transformed into a global Earth coordinate system for neutralizing orientation changes [17].

Under those circumstances, this research assumed the orientations of sensors are known in advance. In case the orientations are unknown, there are a number of suitable algorithms that can be employed to determine these orientations from raw readings [18,19]. The primary aim of this study is discriminating the sensor positions with orientation-dependent features from sensor axes and orientation-independent features from sensor magnitude. By applying feature selection techniques, we not only filter out highly correlated features but also select the most relevant features for target classifications. Finally, the hierarchical classification is built from the optimized local classifications. Particularly, all the on-body sensor positions are divisible into sides and segments, which are referred as local classifications and analyzed in latter sections. The selected algorithms for local classifications are later combined for solving the general problem. The contributions of this research are mentioned as the following:

- We address the importance of feature selection for sensor position classification. Good features have a high impact on the final performance of a model and vice versa. However, selecting a list of features for a classification task is complicated because of the large number of possibilities in combination. Therefore, the features should be selected following a certain procedure for not only reducing dimensionality but also increasing the final performance.
- This research investigates the impact of fractal dimension (FD) as a feature for classification. The FD is known as a feature that highlights the chaotic characteristic of data. In this paper, the usefulness of FD has been proven for discriminating different signals.
- We also consider the impact of data scaling and feature scaling in this research. Performing good prepossessing techniques is a prerequisite for great and interpretive results.
- We perform optimization of classifiers for each local discrimination. These discriminations provide a better solution for practical applications. For example, the classifications of arm- and hand- sides are necessary for human–computer interaction using arm movements.

The other sections of the paper are organized as follows: Section 2 presents a literature survey of on-body position classification. Section 3 describes our approach for solving the problem, followed by the results and performance evaluation in Section 4. Discussions based on the results are presented in Section 5. Finally, Section 6 draws the conclusions of this article.

## 2. Related Works

The sensor or smartphone placement identifications have been a research topic for different context-aware applications in ubiquitous computing communities. Table 1 summarizes a comparison of the related works on sensor positioning in terms of target positions, the number of subjects, sensor types, classifiers, evaluation methods and performance.

The target positions, which correspond to the location of wearable devices or a smartphone, range from head to foot. A wearable device is typically attached firmly to the subject’s body, but a smartphone is usually held in hand or kept in a pocket or a bag. Given a list of target positions, the studies listed in Table 1 proposed robust solutions for classifying as many positions as possible from this list. However, for more straightforward situations, such as differentiating between the hand and the thigh positions, these robust solutions might seem computationally expensive. Using various sensors on a body segment are proposed in the work [20], which shows that it is possible to distinguish between sensors placed on the same body segment using orientation data.

Depending on the target positions, the relevant features may vary as listed in Table 2. Getting relevant features from the raw signal is a two-step process. In the first step, the important features are extracted from the raw signal; this affects the discrimination ability of algorithms. In the second step, some of these extracted features are selected and combined to establish derived features, which are informative and non-redundant in comparison with the extracted features alone. The advantage of using this two-step process is to reduce the training time and improve the interpretability and performance of algorithms. In the following section, we will describe the feature extraction and selection approaches used in this experiment.

Once the target positions have been defined and pertinent features are prepared, the next step is implementing suitable machine learning algorithms. Table 1 summarizes a comparable performance for sensor position recognition of popular machine learning approaches. The most common of these algorithms are Naive Bayes (NB), Pruned decision tree (J48), k-nearest neighbor (kNN), support vector machine (SVM).

For validation, the data is separated into training and testing sets based on two different approaches: leave-one-subject-out (LOSO) and n-fold cross-validation (nCV). In the LOSO approach, a model is trained using data from all the subjects except one, which is used for testing the model later on. This procedure is repeated for each subject, meaning that the data from each subject is left out in each validation. This kind of cross-validation is useful for processing human motion data because it generalizes the trained model to the data from new subjects. Alternatively, the nCV splits data into n equal size sets. One set is used for testing and the remaining sets is used for training. The nCV process is then repeated n times, with each of the n sets used exactly once for testing. The results of both validation approaches are averaged to produce a single estimate. This approach is crucial for selecting the right machine learning algorithms because it measures the classifier performance for comparing one machine learning algorithm with others.

## 3. Methodology

**Figure 1:**
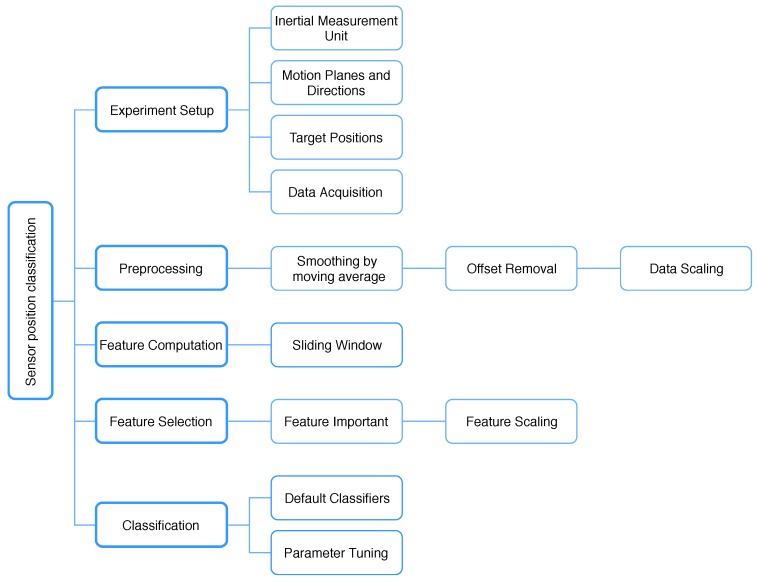
Overview of sensor position classification.

Figure 1 illustrates the data flow from the experimental setups to the classifier optimization. The specifications of the inertial measurement unit (IMU), motion planes and directions, target positions and data acquisition are considered in the experimental setup. In the preprocessing step, the data is smoothed by the moving average technique and having its offset removed. The data is then scaled to an appropriate range for further analysis. After segmenting the data into smaller windows, features are computed for highlighting several data characteristics. From these features, we select appropriate features based on their importance. The default and tuned classifiers are compared to optimize the set of parameters for better results.

### 3.1. Experimental Setups

#### 3.1.1. Inertial Measurement Unit

**Figure 2:**
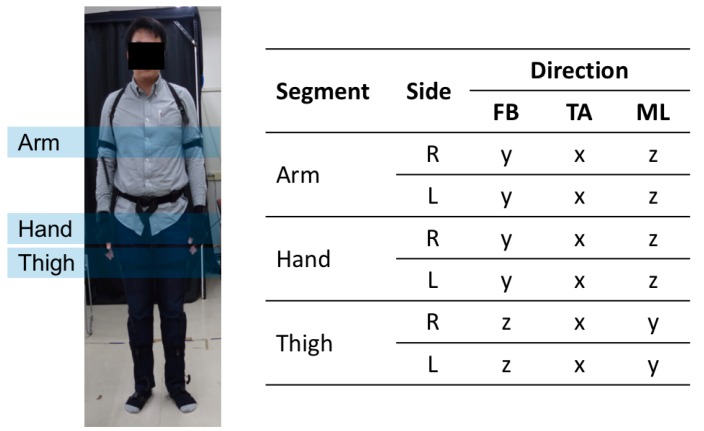
Target positions used in this study, FB: forward and backward, TA: toward and away, ML: medial and lateral.

As the performance of accelerometers and gyroscopes has been improving; the sensor-based motion capture system is being widely used in current research. In our experiment, data was collected using a 3D motion capture system manufactured by NoiTom [28]. The system is based on inertial measurement units (IMUs), each of which includes a 3-axis accelerometer, a 3-axis gyroscope, and a 3-axis magnetometer. The default sampling frequency was 120 Hz. Only the accelerometers (unit is g) and the gyroscopes (unit is radian per second) were used for sensor position classification. Data from all positions was recorded simultaneously and sent to a computer running the Axis Neuron software. The outputs of the software were linear acceleration and angular velocity of body segments with local axes. These local axes are visualized in the Figure 2.

#### 3.1.2. Motion Planes and Directions

The relationships between different parts of the body can be described using motion planes such as sagittal, frontal, and transverse [29]. Firstly, the sagittal plane divides the body into left and right parts and the movements in forward and backward (FB) directions occur here. Secondly, the frontal plane separates the body into front and back parts. The movements toward or away (TA) from the center line of the body take place in the frontal plane are called adduction and abduction, respectively. Finally, the transverse plane divides the body into upper and lower parts and contains movements in medial and lateral (ML) directions, correspondingly. The sensor coordinates at each body position are relabeled following the above motion directions in Figure 2.

#### 3.1.3. Target Positions

Six common positions shown in Figure 2 are selected as the classification targets: (1) right arm; (2) left arm; (3) right hand; (4) left hand; (5) right thigh; (6) left thigh. They are all on-body positions where people carry their smartphone during walking [30]. In the rest of this paper, the term ‘side’ refers to the right or the left body portions, the term ‘segment’ refers to the arm, the hand or the thigh of a subject, and the term ‘position’ refers to any of the target positions.

#### 3.1.4. Data Acquisition

As can be seen from Table 1, walking is the most common physical activity for sensor position classification based on the fact that walking is the most frequent activity throughout a day. Moreover, walking also provides gait patterns which play an important role in detecting health problems [7,31]. In our research, ten subjects (six male, four female) have heights of 161.4±6.1 cm, weights of 59.5±7.2 kg, and age ranging from 22 to 27 years old. Additionally, all subjects are right hand-preferred and have no asymptomatic gait. They were asked to stand still for ten seconds before and after walking for the ease of identifying the start and stop of the activity. They walked straight at their preferred speed for five meters and then turned around to walk back, repeated the process for two minutes. During walking, the subjects were allowed to turn freely sideways. Although the medial and lateral movements generate a comprehensive dataset, they may also lead to misclassifications.

### 3.2. Preprocessing

**Figure 3:**
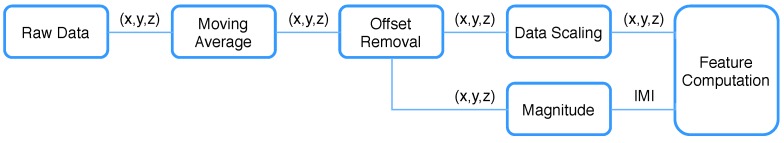
The flows of data in preprocessing stage.

The flow of preprocessing steps is shown in the Figure 3. Each axis of the sensors was smoothed by the moving average technique before having its offset removed. In the next step, the magnitude was calculated from three axes of each sensor. The sources for feature computation include three scaled sensor axes and its magnitude. The three axes are normalized into the range of [−1, 1] because they are equally important to the classification. In contrast, the magnitude of sensors should not be scaled because the dominant side muscles are significantly stronger than the other side [32]. It is also well known that the body is asymmetrical and the symmetry assumption only serves the purpose of simplification [33]. If the magnitude is scaled, the sensors from both sides of a segment are treated equally, which dissents the earlier findings. Therefore, in this experiment, the sensor magnitudes were not scaled regarding the above references.

#### 3.2.1. Moving Average

To eliminate noise and generate a smooth trend, the data is filtered using the 10-point moving average technique, which takes the average of the ten most recent consecutive values as a new sample point. This filtering is commonly used in human activity recognition [34,35,36] and gait analysis [37]. All data values are given equal weight for calculation.

#### 3.2.2. Offset Removal

Theoretically, when a subject is standing-still, all sensor readings should be zero. However, in practice, these values may vary from zero, which generates an offset. For removing this offset, the average sensor values during standing are subtracted from the sensor values during walking. Similar studies applying this offset removal technique can be found in [38,39].

#### 3.2.3. Min-Max Normalization

**Figure 4:**
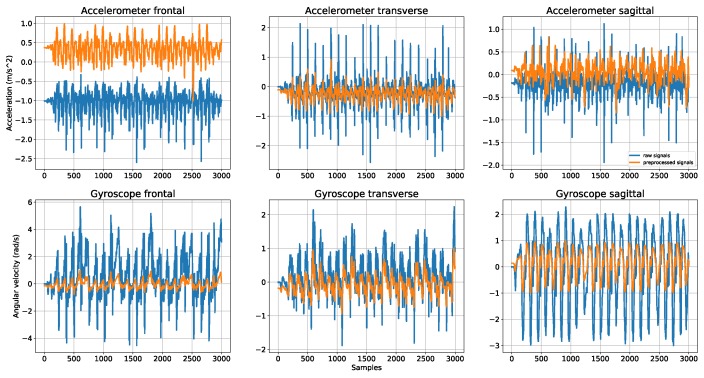
First 25 s of raw and the preprocessed data from the accelerometer at right thigh of one subject.

Data scaling is a preprocessing technique usually employed to transform data of each subject in a certain range before feature computation and classification. Many machine learning-based algorithms use data from various sources such as an accelerometer and a gyroscope, which should be normalized for better comparison and improving performance. In this paper, each sensor axis is normalized to the range [−1, 1] using the following formula:

(1) $$x_{norm}=2\frac{x-min_{x}}{max_{x}-min_{x}}-1$$

where *x* is the preprocessed sensor axis and $min_{x}$ and $max_{x}$ are the minimum and maximum values for each axis, respectively. Figure 4 shows the raw and preprocessed data.

### 3.3. Feature Computation

#### 3.3.1. Sliding Window

The impact of window sizes for human activity recognition is presented in the paper [40]. It would be very difficult for classification based on the raw signals. One reason is that it requires a lot of memory and computational cost for classifications. Another reason is that it is difficult for two signals to be identical, even when they come from the same subject performing the same activity. Under these circumstances, one popular solution is computing features from each time window. The computation provides quantitative measures for comparing two signals.

**Figure 5:**
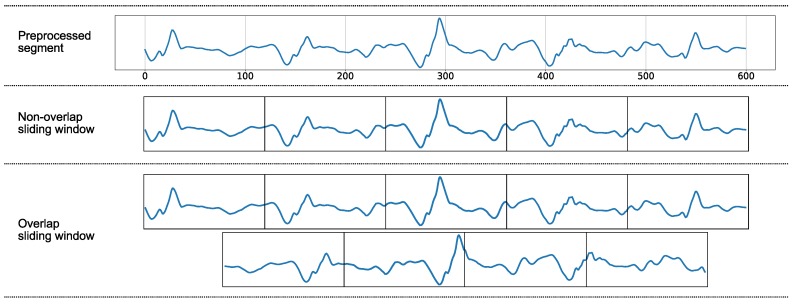
An overlapping sliding window creates more patterns than a non-overlapping sliding window.

Figure 5 illustrates that an overlapping sliding window generates more patterns than a non-overlapping sliding window. Particularly, for each 600 data points (corresponding to 5 s), the non-overlapping sliding window technique provides 5 patterns while the overlapping sliding window technique yields 9 patterns. Additionally, the overlapping sliding window has been shown to be suitable for various recognition applications [23] and provided higher accuracy than non-overlapping window [41]. For this reason, in this research, we applied the sliding window technique with a 50% overlap to avoid missing events and activity truncation. Short time windows may not provide sufficient information for classifications, while long windows may contain turns within a single time window. The small windows (2 s or less) have been proved to give the most accurate performance for human activity recognition [40]. However, for sensor position detection, the longer window size are often selected such as 4 [1], 5 [24], 5.12 [21], 10 [22], 10.24 [21] and 20.48 [21], 45 [26] s, respectively. Our selected window size is 5 s as this period is sufficient to capture the repetitive patterns of sensors during walking.

#### 3.3.2. Signal Characteristic Measures

It is possible to highlight sensor signals by several characteristics such as centrality, position, dispersion, distribution, chaos and energy. By assuming readers are already familiar with the popular features, this research briefly introduces them and moves into details for other features.

Centrality measures includ the computation of mean and root-mean-square (RMS). The position characteristic of data is presented as percentiles. In this experiment, we use values at 25th, 50th and 75th percentiles as features and denote them as ‘per25’, ‘per50’, ‘per75’, respectively. The standard deviation, variance, minimum, maximum, range, interquartile range (IQR) presents the dispersion features of data. The measures of asymmetry and peakedness in data distribution are skewness and kurtosis, correspondingly. The energy feature was used to discriminate motions in different directions. The entropy is a well-known feature for quantifying the chaos of data. We add a fractal dimension (FD) into consideration and compare it with entropy regarding several classifications. The differences between them will be discussed more detail as the following.

- **Entropy**: Entropy refers to the disordered behavior of a system and originates from the second law of thermodynamics. The entropy of a system is always increasing as the system evolves and becomes constant when the system reaches its equilibrium. However, when the entropy of a system decreases, that means the system is affected by external factors. The Shannon entropy expresses the average information content when the value of the random variables is unknown.
  (2) $$entro=-\sum_{j=1}^{m}p_{j}\log_{2}p_{j}$$
  where *m* is the number of bins of the histogram and *p* is the probability mass function of *i*th bin.
  The data from motion sensors is a continuous signal, which needs to be converted into a histogram for calculating Shannon entropy. For each window, the entropy is calculated using different bin sizes of the histogram such as 10, 20, 30, 40, 50 and denoted as ‘entro10’, ‘entro20’, ‘entro30’, ‘entro40’, ‘entro50’, respectively.
- **Fractal Dimension (FD)**: Fractal is an object or a signal that has repeating patterns and a similar display at both the micro-scale and the macro-scale. A FD is used to measure the fractal characteristics and considered as the number of axes needed to represent an object or a signal. In Euclidean space, a line has one dimension while a square has two dimensions. However, a curved line in a plane is neither one- nor two-dimensional because it is not a line and does not move in two directions. The FD of a curved line is an integer between 1 and 2. If the FD is approaching 1, it means that the curved line is becoming a straight line. In contrast, if FD is approaching 2, it illustrates the ability to cover a square of the curved line. Higuchi’s algorithm [42] is often used to estimate the FD. Although this algorithm sensitive to noise, it yields the most accurate estimation for FD [43].

The first step for computing the FD of a window with size *N* requires the computation of smaller segments called $X_{k}^{m}$. For a given integer *k* (*k* ≥ 1), the window is divided into *k* segments and their lengths are calculated as follows:

(3) $$L_{m}\left(k\right)=\frac{(\sum_{i=1}^{\mathrm{int}(\frac{N-m}{k})}|X\left(m+ik\right)-X\left(m+\left(i-1\right)k|\right)\frac{N-1}{\mathrm{int}(\frac{N-m}{k})k}}{k}$$

where *m* is an integer smaller or equal to *k*, the term $\frac{N-1}{\mathrm{int}(\frac{N-m}{k})k}$ represents the normalization factor for the length of each segment. The length of the curve corresponding to the value *k* is defined as the average values of *k* sets of $L_{m}\left(k\right)$. If $<L\left(k\right)\propto k^{-D}>$, then the signal is a fractal with dimension D. In this experiment, *k* values are selected from 2 to 10, which represent the similarity between the big segments and the original signal, and the similarity between the small segments and the original signal, respectively.

We also considered using cardinality [44] as another feature for classifications. The addition of this feature requires the information of sensor resolution for a better understanding the feature impact. However, this specification is not published by the motion sensor system manufacturing company. Therefore, it is left open for future investigations.

### 3.4. Feature Selection

Feature selection is a technique for reducing the computation time and improving the classification performance. Although feature selection and dimensionality reduction both reduce the size of the dataset, there is one key difference: the technique of feature selection yields a subset of the original feature set, but this result is not hold for dimensionality reduction. For example, principal component analysis reduces the dimensionality by generating new synthetic features from the original feature set and then discarding the less important ones. In our research, we use feature selection because it selects features that give a good result while requiring fewer data.

A comprehensive review of feature selection is presented in [45]: three common techniques are filtering, wrapper and embedded methods. The filtering methods is considered as a kind of preprocessing because they are applied before the classification to filter out less relevant features. As the main role of a feature is to provide useful information for discriminating classes, the wrapper technique employs different search algorithms to find a subset of features that gives the highest classification performance. A tree structure is used to evaluate different subsets of the given feature set. However, this search grows exponentially as the size of the feature set increases and exhaustive search methods become computationally intensive for large datasets. The embedded methods select features as a part of the training process without splitting the data into training and testing sets. Embedded methods reduce the computation time required for reclassifying different subsets, which is what happens in the wrapper methods. The objective function of the embedded method is designed for maximizing the mutual information between the features and the class output while minimizing the mutual information between the selected features and the subset of selected features.

In this research, we filtered highly correlated features (correlation coefficient ≥ 0.9) then employed the novel technique of recursive feature addition (RFA), which is a forward feature selection method. The RFA starts with an empty feature set and keeps adding one feature at a time until an ending criterion is met. The method utilizes an eXtreme Gradient Boosting (XGB) classifier, which will be introduced in more detail in the next section, as a core classifier to rank the features. Based on the ranked features, several classifiers are employed for comparison. These classifiers will be discussed in more details in the next section. The general approach of this feature selection method is described as the following steps:

**Step 1:** The highly correlated features are removed to reduce the size of feature space.

**Step 2:** Rank the features according to their importance derived from XGB classifier.

**Step 3:** Build machine learning models with the first-ranked feature. The feature selection is optimized for each classifier, which goes through the leave-one-subject-out (LOSO) cross-validation. The average of F1-score is then calculated as the initial valuation.

**Step 4:** Add the next ranked feature and rebuild the machine learning algorithms utilizing the additional feature with all the features from the previous step.

**Step 5:** Calculate the performance metric F1-score for the additional feature with LOSO cross-validation.

**Step 6:** If the F1-score increases by a threshold of 0.01, then that feature is important and should be kept for the final classification. Otherwise, the additional feature is removed from the final feature list.

**Step 7:** Repeat steps 4–6 until all the features are evaluated the corresponding performance using F1-score.

This method is faster than the wrapper one because firstly, it ranks and then adds one feature at a time based on its importance. The wrapper method is computationally intensive and time-consuming since it requires constructing all candidate feature subsets [45]. In addition, our method is similar to the embedded technique as it makes classifier-dependent selections, which might not work with other classifiers.

The next important step is feature normalization. In the same way of data normalization, the selected features may have different dynamic ranges. Particularly, features with large ranges have more weight than those with smaller ranges for the distance-based classifications. As a result, normalizing is required to make the selected features have approximately the same effect in the computation of similarity. In this work, they were scaled to the range [0,1], which is recommended by [46].

### 3.5. Classification

We provide here a brief introduction to five commonly used classifiers in the literature that are applicable for classifying human activity. These are logistic regression (LR), k-nearest neighbor (kNN), decision tree (DT), support vector machine (SVM) and extreme gradient boosting (XGB) classifiers.

- **Logistic Regression (LR)**: The LR is a machine learning classification algorithm that is used to predict the probability of a categorical dependent variable. To generate probabilities, logistic regression uses a sigmoid function that gives outputs between 0 and 1 for all values. In multi-class classification, the algorithm uses the one-vs-rest scheme.

The implementation of logistic regression in scikit-learn can be tuned by changing its parameters ‘penalty’ and ‘C’. The parameter ‘penalty’ takes either ‘L1’ or ‘L2’ value, which indicates a different cost function. The parameter ‘C’ represents the inverse of regularization strength. A smaller ‘C’ value reflects a stronger regularization. In this experiment, the value of C is varied from 0.1 to 5 with the steps of 0.1.

- **k-Nearest Neighbor (kNN)**: The kNN is a machine learning approach that uses distance measures between data for classification. Particularly, given a new sample, the approach takes the major votes of the *k* closest samples from the training set to assign a class to the unknown sample. The parameters that affect the algorithm performance are the number of nearest neighbors, the weights of each value and the distance calculation methods. The number of nearest neighbors is varied from one to ten samples. The weight is alternated between ‘uniform’ and ‘distance’: ‘uniform’ means that all samples are weighted equally and ‘distance’ means that the sample weights are calculated to be the inverse of their distances. The parameter ‘p’ has two values 1 and 2, which are equivalent Manhattan distance and Euclidean distance, correspondingly. Additionally, the ‘algorithm’ parameter refers to computing methods of the nearest neighbors and has several options such as ‘auto’, ‘ball_tree’, ‘kd_tree’, ‘brute’.
- **Decision Tree (DT)**: The DT is a predictive model based on a set of hierarchical rules to generate outputs. It is a common approach for classification because it has high interpretability for human understanding.

The DT model is influenced by several parameters. The first parameter is ‘criterion’, which takes either ‘gini’ or ‘entropy’ criteria to measure the quality of a split. The ‘gini’ selection stands for using Gini impurity, while ‘entropy’ criteria use information gain for splitting data. The ‘entropy’ option requires more time to compute than ‘gini’ option. The second parameter is ‘max_depth’, which is used to indicate the maximum tree depth. This parameter is ranged from 1 to 32 to seek for an appropriate value for the best classification. The third parameter is ‘min_samples_split’, which refers to the minimum ratio of samples required to split an internal node. In this experiment, this parameter is a float ranged from 0.1 to 1 with the steps of 0.1. The final parameter ‘min_samples_leaf’ stands for the minimum ratio of samples required to be at a leaf node. This parameter must be at least 1 (by default classifier) as an integer or (0,1] as a float number. In this experiment, it ranges from 0.1 to 0.5 with steps of 0.1 in this experiment.

- **Support Vector Machine (SVM)**: The SVM classifier is one of the most popular machine learning algorithms used in classifications. It is based on finding a hyperplane that best divides a dataset into two classes. The SVM classifier has the key parameters of ‘C’, ‘gamma’ and ‘kernel’. The parameter ‘C’ indicates the large margin for splitting the data into two parts, and vice versa. The parameter ‘gamma’ defines how far the influence of a single training example reaches. In this experiment, both the ‘C’ and ‘gamma’ parameters are varied from 0.1 to 5 in steps of 0.1. The parameter ‘kernel’ has several options such as ‘linear’, ‘poly’, ‘rbf’, ‘sigmoid’ corresponding to different activation functions.
- **Extreme Gradient Boosting (XGB)**: The XGB classifier is a highly flexible and versatile technique that works through most classifications. In the classification area, weak and strong classifiers refer to the correlations of outputs and targets. Boosting is an ensemble method that seeks to create a strong classifier based on weak classifiers. By adding classifiers on top of each other iteratively, the next classifier can correct the errors of the previous one. The process is repeated until the training data is accurately predicted. This approach minimizes the loss when adding the latest classifier, and the models are updated using gradient descent, leading to the name “gradient boosting”.

The XGB classifier is tuned by the following parameters. The parameter ‘eta’ represents the learning rate; it was varied in from 0.1 and 1 with steps of 0.1. The maximum depth of a tree is controlled by the parameter ‘max_depth’, which was varied from 3 to 10. The ratio of the sub-sample and the sample for each tree is reflected in the parameter ‘subsample’, which was varied from 0.5 to 1.0 in steps of 0.1. The loss function is selected by the ‘objective’ parameter. It has several options such as ‘reg:linear’, ‘reg:logistic’, ‘binary:logistic’, ‘binary:logitraw’, which stand for linear regression, logistic regression, logistic regression for binary classification (output probability), logistic regression for binary classification (output score before logistic transformation), respectively.

### 3.6. Validation

It is important to choose an appropriate validation method for the selected classifiers. One approach is cross-validation, where the inputs are divided into training and testing sets. The testing set is used to validate the model generated by the training set. In our research, we applied leave-one-subject-out (LOSO) cross-validation, wherein each subject is used for testing and the other subjects are used for training. This process is repeated until all the subjects are tested and applied to every classification.

### 3.7. Hierarchical Classification

**Figure 6:**
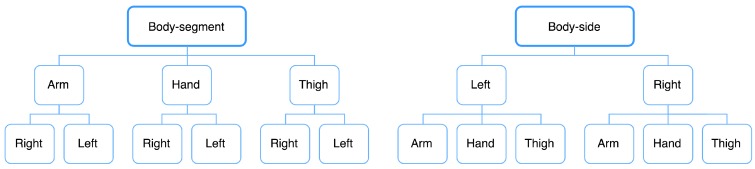
The hierarchical classification body-segment approach (**left**) and body-side approach (**right**).

The advantages and disadvantages of hierarchical classification methods are deeply discussed in this article [47]. The traditional classification algorithm is considered as a flat classification approach as it ignores the class hierarchy and parent-child class relationships, typically predicting classes at the leaf nodes. Another approach comprises grouping the data in a hierarchical manner, where similar classes are grouped together into meta-classes, resulting in a hierarchical classifier. In our research, we applied a local classifier approach as illustrated in Figure 6, which makes use of local information and explained in the below sections.

#### 3.7.1. Node Classification

It is necessary to find local information at each of the seven nodes. The arm-side, the hand-side, the thigh-side and the body-side require binary classifications as they need to distinguish between the data from the right and the left side of the body. The right-segment, left-segment and body-segment are three-class classifications as they are the discrimination of the arm, hand and the thigh segments. We optimized these classifications following the processing steps outlined above before being forwarded to the hierarchical classification.

#### 3.7.2. Body-Segment top-down Approach

In this approach, the body-segment is classified as the first node. The first output layout contains the arm, hand, and thigh data. The optimized models from node classification are used for discriminating the right and the left sides at the arm, hand and thigh segments.

#### 3.7.3. Body-Side top-down Approach

This approach takes body-side classification as the priority. The outputs of the first classification are labeled as right- and left-segments. For each segment, a three-class classification is carried out to determine whether it is an arm, hand or thigh position.

## 4. Results

This section presents the results of proposed classification. Firstly, the default classifiers were used to find an appropriate window size and feature sets for classification. Secondly, the parameters of these classifiers were tuned to find an optimal combination of parameters. Finally, a hierarchy classification was implemented to detect the sensor positions.

### 4.1. Body Part Classification

Table 3 displays the result of default and tuned classifiers for body-side and body-segment classifications. According to this table, tuning parameters of classifiers improves the performance in many cases. The gyroscope provide better performance than the accelerometer regardless classifiers. For body-segment classification, the F1-score is 0.96 using gyroscope sensor while it is 0.89 using the accelerometer. A similar observation can be made for body-side classification, the best performance using the gyroscope is 0.83, which is higher than 0.3 in the case of the accelerometer. The parameter tuning improves the SVM classifier by 0.3 for body-segment and 0.2 for body-side classification, respectively. Other cases the performance is slightly improved by 0.1 such as LR, kNN, XGB classifiers. However, tuning parameter decreases the performance of DT classifiers. The classifiers, that provide similar or equal performance, are compared in the term of their processing time before being implemented in the hierarchical classification. For example, in body-segment classification, the LR, SVM and XGB classifiers provide similar performance from which their processing time will be compared in the later section.

### 4.2. Body Segment Classification

The Table 4 presents the performances of default and tuned classifiers for segment discriminations on both sides of the body. The segment classification of the accelerometer data on the left side has best performance with 1.00 F1-score using LR, KNN and SVM classifiers, alternatively. On the other hand, the gyroscope-based left-segment classification using SVM is better than other classifiers. For right-segment classification, the best performance is 0.97 using features from the gyroscope for either LR, SVM or XGB classifiers. The accelerometer-based discrimination also provide high result (0.95). Although DT classifiers is improved when tuning its parameter in this case, its performance is not good as other classifiers. The accelerometer-based left-segment and gyroscope-based right-segment classifications require further comparison in the term of processing time using different classifiers.

### 4.3. Body Side Classification

The discrimination of body sides at each segment are displayed in Table 5. The first impression in the section is the absolute classification (1.00) of right- and left-hand sides using either the accelerometer or the gyroscope. Furthermore, the arm-side classification also provides high result (0.99) regardless classifiers. However, the processing time of these classifiers should be compared to select the most appropriate one for the hierarchical classification. For thigh-side classification, the performance using the gyroscope is slightly better than the accelerometer (0.89 and 0.88).

### 4.4. Tuning Parameters of Classifiers

Table 6 shows the processing time of classifiers that provide the same performance in previous sections. The number of samples is rounded for comparison convenient. The classifications was run on a MacBook Pro with 2.0 GHz Intel Core i5 CPU and 16 GB of RAM with Python version 3.6.5. The first and important observation from this table is that for a specific classification, several classifiers provide a same performance with different processing times. In other words, the processing time is highly dependent on the classifiers and the number of sample. For example, using the LR classifier, the processing time is decreased approximately 7 times when the sample size is reduced one half (2500 sample for body-segment classification and 1200 samples for left-segment classification). However, with the same reduction of sample sizes, the processing time of SVM classifier is decreased about one half (from 475 to 272 ms). Similar observation can be seen for XGB classifier for gyroscope-based body-segment and right-segment classification. Although the most time-efficient classifier is varied for different classifications, the XGB is the most time-consuming classifier.

Under those circumstances, for body-segment classification, the SVM and kNN classifiers are selected for classifying features from the accelerometer and the gyroscope, respectively. The kNN classifiers is suitable for discriminating segments on the left body side because of its fast processing time. For the same reason, the LR classifier is selected for right-segment and arm-side classifications. Correspondingly, the accelerometer-based features from the hand sides are instantly discriminated using the kNN classifier. Finally, the DT requires less time than other classifiers for hand-side classification using gyroscope.

### 4.5. Hierarchical Classification

We present here results from a hierarchical classification built from the optimized local classifiers. From a total of 112 computed features, useful features are selected for each target classification. We compare the body-segment and the body-side top-down approaches. In the body-segment top-down classifications, all positions are first classified based on body segments. For each predicted segment, right and left sides are classified based on the optimized classifiers in Table 7 for the accelerometer and Table 8 for the gyroscope. The corresponding selected features are also presented in these tables. Equally important, the related classifier parameters are presented in the Table 9 and Table 10 for the accelerometer-based and the gyroscope-based classifications.

**Figure 7:**
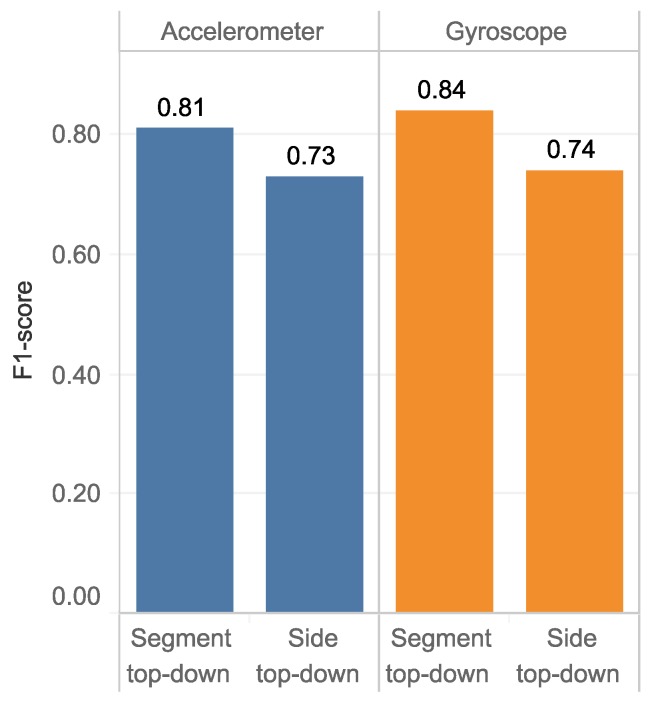
The comparison of final results based on different hierarchical classifiers.

The final results of the hierarchical classification are displayed in Figure 7. The body-segment top-down classification provides the best performance using the gyroscopes (0.84) and the accelerometers (0.81), correspondingly. Similarly, in the side top-down classification, the performance based on gyroscope is slight better than accelerometer (0.74 and 0.73, respectively).

## 5. Discussions

The Table 7 and Table 8 present necessary features for classifications. There are 112 features for each sensor (28 feature for each axis and magnitude). Therefore, feature selection helps reduce the number of features used for classification as well as improve the performance. If a specific application requires an answer for the questions “where is the sensor?”, it is required totally 19 features for the accelerometer or 23 features for the gyroscope, correspondingly. For a specific human-computer interface application demands the body side detection, the number of features when using the accelerometer is reducible to 4 or even 1 for arm-side and hand-side classifications, respectively. The proposed features in this research are based on the accelerometer and the gyroscope. It is important to mention that ‘Outwalk’ is a standardized protocol for measuring 3D kinematic data [48,49]. The findings in [50], which confirmed that the wrist angle is more variable than the hip angle, and other papers using orientation-related features [51,52] have proved their potential in the sensor position classification. The combination of orientation-dependent and orientation-independent features is worthy of further study.

The fractal dimension (FD) and entropy are critical features for characterizing the roughness of signal. However, it is apparent from both the Table 7 and Table 8, the FD feature outperforms entropy for sensor position classification. The entropy feature is useful for discrimination in the cased of the gyroscope-based body-side and arm-side classifications. Different from entropy, the FD is used for most of the classifications and contributive to the final performance.

**Figure 8:**
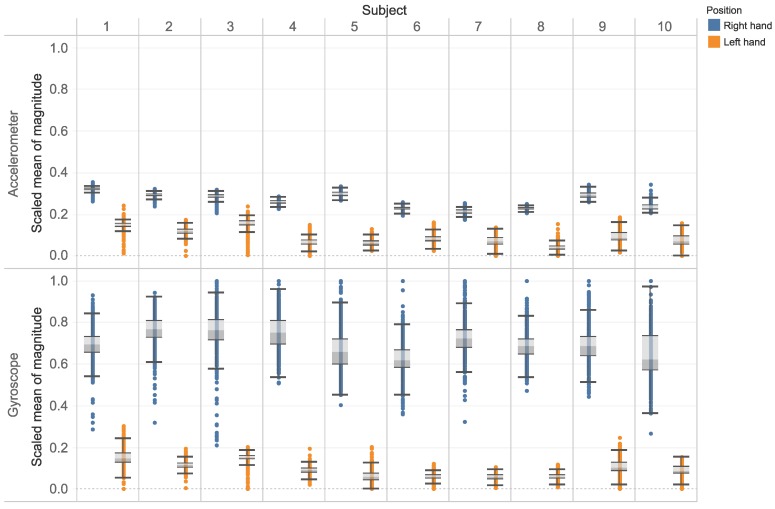
The boxplot for hand sides of all subject using accelerometer and gyroscope.

The Figure 8 shows the boxplots of the right and left hands for all the subjects. Although there are ten subjects participated in this experiment, the result shows that this is a promising approach for on-body sensor classification. Furthermore, this result confirm the finding that for the right hand-preferred people, the dominant hand is stronger than the non-dominant hand. As the result, in this case, the right hand generates greater acceleration and angular velocity than the left hand. However, as stated in the work [32], there is no significant differences were observed between dominant and non-dominant hands in left-handed participants. Therefore, further investigation on the left-handed participants should be carried out in the future together with the increase of subjects for generalizing the conclusion.

This experiment requires subjects starting from a static movement and omits the calibration procedures. For implementing in practise, in addition with feature enhancement, more conditions should be considered. For example, this work should be combined the calibration under different walking conditions [53] or auto-calibration methods [54,55]. These combinations enables the user starts from arbitrary movements ensuing the flexibility of the application in practise.

The impact of window segmentation is ignored in this paper. However, it should be considered in another research with different window sizes or using adaptive sliding window. For instance, the previous offline studies showed promising performance when using adaptive window to predict the user intent during ambulation [56]. The adaptive window segmentation also has benefits in transition motions [57] during continuous activities. Moreover, it is useful for handling with uncertainty from the environment [58,59].

Tuning parameter should be perform because of its usefulness in seeking the appropriate set of parameters to improve the classification result. The well-known example of parameter tuning is finding the number of *k* when using kNN classifier [60]. Specifically, if k is too small, the result may sensitive to noises or outliers. On the other hand, if k is too large, there is a chance that the neighbors are dominated by many points from other classes. Similarly, other classifiers are parameter-dependent, which should be adjusted for desired outcomes. In this research, an additional feature are appended to the final list if it improves the performance throughout LOSO cross-validation, which demonstrates the stability for a new subject. Therefore, tuned classifiers for that feature space also stable for a new subject.

This research has both advantages and disadvantages for real-time application. The practical online applications typically focus on specific classifications, which is the aim of this research. For example, discriminating arm- and hand-side for human–computer interaction. These classifications are optimized in the terms of features and classifiers with the fast performance in this paper. Another advantage of this research is that data is well preprocessed using a simple approach. As a result, a few features are required for classification such as using 10 features from accelerometer to detect sensor segments. The limitation for online implementation is the use of long window size (5s), which can be overcome by combining with other above-mentioned studies. The sampling frequency also important for long-time operation. This issue should be considered in future work for investigating the impact of sampling frequency on sensor position classifications.

## 6. Conclusions

In our approach, we applied hierarchical classification on a dataset of ten healthy people walking and turning around for two minutes. The subjects were allowed to turn freely in the medial and lateral directions for generating a comprehensive dataset. During walking, motion information was collected at six different positions. Several classifications at the local level were optimized and combined together to solve the general problem. Specifically, classification was carried out based on seven distinct categories: body-side, body-segment, left-segment, right-segment, arm-side, hand-side and thigh-side. Each classification was optimized by feature selection for different classifiers. These models later are combined to perform a 6-class classification. The following are the main results of our research:

- Together with data scaling, features scaling should be executed to normalize selected features for better classification results.
- For representing the chaotic characteristic of data, FD has outperformed entropy feature in most of particularly classifications.
- It is possible for several classifiers to have same performance. Nevertheless, these classifiers requires a different amount of time for processing data. Therefore, the system performance and processing time should be examined together for a robust implementation.
- Local information can be used to solve specific problems: for example, discriminating between arm-side and thigh-side positions gives a better performance with F1-scores of 0.99 and 0.88, respectively. This indicates that the popular features of well preprocessed data are enough to discriminate among these classifications.
- It was found that, the body-side top-down approaches have a relative performance for both the accelerometer (0.73) and the gyroscope (0.74) for the 6-class classification. However, in body-segment top-down approach, the average F1-score increases to 0.81 and 0.84 for the accelerometer and the gyroscope, respectively. However, since the gyroscope is energy-hunger than the accelerometer [21], for long-time operation, we recommend using the accelerometer.

As future work, we plan to generalize these findings on other datasets and combine with aforementioned approaches. Other related datasets have been collected all over the world, which generate a comprehension on ethnicity, gender, age and health status. For example, there is a different walking style between western and Asian people, man and woman, young and elder people, normal people and patients. It would also be interesting for deeply considering transition states such as turning when changing direction. The performance of these findings will be evaluated in context-aware applications.

## Figures and Tables

**Table 1:** Related works on sensor position classification.

Ref.	Target Positions (Total Number)	Activities (Total Number)	Sensor(s)	Subjects	Feature Selection	Classifier(s)	Evaluation	Performance
[1]	Chest, coat pocket, thigh pocket, and hand (4)	Walking, standing and other activities (6)	Linear Accelerometer Accelerometer Gravity Gyroscope	15	3D to 2D projection	DT SVM NB	n-fold	93.76 (DT) 94.22(SVM) 84.71(NB)
[21]	Neck, chest, jacket, trouser front/back, 4 types of bags (9) Merged: “trousers”, “bags” (5)	Walking, standing and other activities (7)	Accelerometer	20	Correlation-based	DT NB SVM MLP	LOSO n-fold	80.5 99.9 85.9 (merged)
[22]	Ankle, thigh, hip, upper arm, wrist (5) Ankle, hip, wrist (3) Ankle, wrist (2)	Walking, standing and other activities (28)	Accelerometer	33	none	SVM	LOSO	81.0 (5-class) 92.4 (3-class) 99.2 (2-class)
[23]	Dataset 1: backpack, messenger bag, jacket pocket, trouser pocket (4)	Walking, standing and other activities (5)	Linear Accelerometer Accelerometer Gravity	10	WEKA machine learning tool-kits	kNN DT RF MLP	LOSO	76.0 (Dataset1)
	Dataset 2: trouser pocket, upper arm, belt, wrist (4)	Walking, standing and other activities (6)	Linear Accelerometer Accelerometer Gravity Gyroscope Magnetic	10				93.0 (Dataset 2)
	Dataset 3: backpack, hand, trouser pocket (3)	Walking, standing and other activities (9)	Accelerometer	15				88.0 (Dataset 3)
[24]	Hand holding, talking on phone, watching a video, pants pocket, hip pocket, jacket pocket and on-table (7)	Walking, standing and other activities (3)	Accelerometer Gyroscope Microphone Magnetic	10	none	DT NB MLP LR	n-fold	88.5 (Accelerometer) 74.0 (Gyroscope) 89.3 (fused)
[25]	Head, upper arm, forearm, waist, thigh, shin (6)	Walking and non-walking (2)	Accelerometer	25	none	SVM	n-fold	89.0
[26]	Head, torso, wrist, front pocket, back pocket (5)	Walking and non-walking (2)	Accelerometer	6	none	HMM	n-fold	73.5
[27]	Pelvis, sternum, head, right shoulder, right upper arm, right forearm, right hand, left shoulder, left upper arm, left forearm, left hand, right upper leg, right lower leg, right foot, left upper leg, left lower leg and left foot (17)	Walking and non-walking (2)	Accelerometer Gyroscope	10	none	DT	n-fold	97.5

DT: Decision Tree; NB: Naive Bayes; SVM: Support Vector Machine; MLP: Multilayer Percentron; kNN: k-Nearest Neighbor; RF: Random Forest; LR: Linear Regression; HMM: Hidden Markov Model; LOSO: Leave-One-Subject-Out.

**Table 2:** Common features for sensor position classification.

Feature	References								
	[21]	[1]	[22]	[23]	[24]	[25]	[26]	[27]	This Work
Mean		x	x	x	x		x	x	x
Standard deviation	x	x	x	x	x		x		x
Variance				x	x			x	x
Minimum	x	x				x			x
Maximum	x	x				x			x
Range									x
Percentile	x								x
Inter quartile range									x
Root-mean-square								x	x
Number of peaks		x					x		
Zero-crossing rate				x			x		
Skewness									x
Kurtosis									x
Entropy									x
Fractal dimension									x
Energy		x							x

**Table 3:** Result of optimized classifiers for body-segment, body-side classifications.

Clasification	Sensor	LR		kNN		SVM		DT		XGB	
		D	T	D	T	D	T	D	T	D	T
Body-segment	A	0.86	**0.88**	0.80	0.81	0.86	**0.88**	0.84	0.76	**0.88**	**0.89**
	G	0.89	0.90	**0.96**	**0.96**	0.93	**0.96**	0.89	0.81	0.95	**0.96**
Body-side	A	0.80	0.80	0.64	0.66	0.79	0.80	**0.82**	0.78	0.68	0.68
	G	0.84	0.84	**0.86**	**0.86**	0.81	0.83	0.83	0.81	0.81	0.81

A: Accelerometer, G: Gyroscope, D: Default classifier, T: Tuned classifier.

**Table 4:** Result of optimized classifiers for left-segment, right-segment classifications.

Clasification	Sensor	LR		kNN		SVM		DT		XGB	
		D	T	D	T	D	T	D	T	D	T
Left-segment	A	**1.00**	**1.00**	0.99	**1.00**	**1.00**	**1.00**	0.92	0.93	0.96	0.96
	G	0.95	0.96	0.98	0.99	0.98	**1.00**	0.95	0.93	0.95	0.96
Right-segment	A	**0.95**	**0.95**	0.93	0.93	0.93	0.94	0.84	0.86	0.90	0.91
	G	**0.97**	**0.97**	0.95	0.95	**0.97**	**0.97**	0.89	0.78	0.95	**0.97**

A: Accelerometer, G: Gyroscope, D: Default classifier, T: Tuned classifier.

**Table 5:** Result of optimized classifiers for arm-side, hand-side and thigh-side classifications.

Clasification	Sensor	LR		kNN		SVM		DT		XGB	
		D	T	D	T	D	T	D	T	D	T
Arm-side	A	**0.99**	**0.99**	**0.99**	**0.99**	**0.99**	**0.99**	0.95	0.95	**0.99**	**0.99**
	G	0.70	0.71	**0.81**	**0.81**	0.66	0.75	0.70	0.79	0.53	0.62
Hand-side	A	0.99	**1.00**	**1.00**	**1.00**	0.99	**1.00**	**1.00**	**1.00**	**1.00**	**1.00**
	G	**1.00**	**1.00**	**1.00**	**1.00**	**1.00**	**1.00**	**1.00**	**1.00**	**1.00**	**1.00**
Thigh-side	A	0.62	0.62	0.51	0.54	0.60	0.67	0.54	0.70	**0.88**	**0.88**
	G	0.82	0.88	0.86	0.88	0.84	**0.89**	0.82	0.88	0.88	0.88

A: Accelerometer, G: Gyroscope, D: Default classifier, T: Tuned classifier.

**Table 6:** Processing time of classifiers that have similar performance.

Classification	Sensor	Classifier	Samples		Number of Features	Processing Time	
			Train	Test		Mean (ms)	Std (ms)
Body-segment	A	LR	2500	300	8	603.0	14.1
		SVM	2500	300	10	**475.0**	**26.6**
		XGB	2500	300	10	4630.0	113.0
	G	kNN	2500	300	8	**142.0**	**4.9**
		XGB	2500	300	4	2530.0	16.0
Left-segment	A	LR	1250	150	7	78.0	0.5
		kNN	1250	150	4	**74.5**	**10.7**
		SVM	1250	150	6	272.0	14.8
Right-segment	G	LR	1250	150	6	**79.3**	**0.7**
		SVM	1250	150	6	268.0	11.2
		XGB	1250	150	6	1210.0	87.0
Arm-side	A	LR	800	100	4	**56.0**	**0.7**
		kNN	800	100	4	59.1	0.2
		SVM	800	100	3	91.7	0.6
		XGB	800	100	4	333.0	2.2
Hand-side	A	LR	800	100	1	84.2	0.1
		kNN	800	100	1	**62.1**	**6.3**
		SVM	800	100	1	86.1	7.5
		DT	800	100	1	74.7	19.3
		XGB	800	100	1	186.0	18.2
	G	LR	800	100	1	64.4	8.24
		kNN	800	100	1	79.0	9.5
		SVM	800	100	1	71.1	7.4
		DT	800	100	1	**61.2**	**4.8**
		XGB	800	100	1	197.0	12.0

A: Accelerometer, G: Gyroscope.

**Table 7:** The performance of selected features and classifiers using the accelerometer.

Classification	Classifier	Feature Types							P	R	F1	Processing Time	
		Centrality	Position	Dispersion	Distribution	Chaotic	Energy	#				Mean (ms)	Std (ms)
Body-segment	SVM	mean-FB mean-TA		std max-TA	kurt-FB skew skew-ML	k-2 k-4-TA k-8-TA		10	0.90	0.88	0.88	475.0	26.6
Body-side	DT	mean mean-FB RMS		std std-TA	skew-ML	k-4		7	0.83	0.82	0.82	204.0	17.3
Left-segment	kNN	mean mean-TA		std		k-4-TA		4	1.00	1.00	1.00	74.5	10.7
Right-segment	LR	mean-ML mean-TA		std max-ML	skew-FB	k-2-FB k-4-FB		7	0.95	0.95	0.95	91.5	12.5
Arm-side	LR	mean-FB mean-ML mean-TA			skew-FB			4	0.99	0.99	0.99	67.4	4.1
Hand-side	LR	mean						1	1.00	1.00	1.00	73.1	13.1
Thigh-side	XGB	mean mean-ML		std-TA	kurt-FB			4	0.91	0.89	0.88	468.0	30.8

FB: forward and backward direction, TA: toward and away direction, ML: medial and lateral direction; #: total number of feature, P: precision, R: recall, F1: F1-score.

**Table 8:** The performance of selected features and classifiers using gyroscope.

Classification	Classifier	Feature Types							P	R	F1	Processing Time	
		Centrality	Position	Dispersion	Distribution	Chaotic	Energy	#				Mean (ms)	Std (ms)
Body-segment	kNN	mean mean-ML		IQR min std-FB std-TA	kurt-FB	k-8-TA		8	0.96	0.96	0.96	158.0	17.8
Body-side	kNN	mean mean-FB mean-TA	per25-FB per75-ML		kurt-FB	entro20		7	0.87	0.86	0.86	146.0	15.3
Left-segment	SVM	mean	per50-FB	std-FB	skew-FB	k-8-TA		5	1.00	1.00	1.00	90.5	7.4
Right-segment	LR	mean mean-TA		min-FB std-TA	skew-ML	k-2-ML		6	0.97	0.97	0.97	84.5	5.0
Arm-side	kNN	mean-FB	per50-ML	min	kurt-TA	k-4-FB entro-50		6	0.84	0.82	0.81	73.5	3.3
Hand-side	DT	mean						1	1.00	1.00	1.00	58.9	7.2
Thigh-side	SVM				skew-FB			1	0.94	0.91	0.89	167.0	6.6

FB: forward and backward direction, TA: toward and away direction, ML: medial and lateral direction, #: total number of feature, P: precision, R: recall, F1: F1-score.

**Table 9:** Parameters of selected classifiers using the accelerometer.

Classification	Classifier	Parameters	Note
Body-segment	SVM	C = 2.0, gamma = 0.5, kernel = ’poly’	Tuned
Body-side	DT	criterion = ’gini’, max_depth = None, min_samples_leaf = 1, min_samples_split = 2, splitter = ’best’	Default
Left-segment	kNN	n_neighbors=5, weights=’uniform’, p=1	Tuned
Right-segment	LR	C = 1.0, penalty = ’l2’	Default
Arm-side	LR	C = 1.0, penalty = ’l2’	Default
Hand-side	LR	C = 1.0, penalty = ’l2’	Default
Thigh-side	XGB	learning_rate = 0.1, max_delta_step = 0, objective = ’binary:logistic’, subsample = 1	Default

**Table 10:** Parameters of selected classifiers using gyroscope.

Classification	Classifier	Paramters	Note
Body-segment	kNN	n_neighbors = 5, p = 2, weights = ’uniform’, algorithm = ’auto’	Default
Body-side	kNN	n_neighbors = 5, p = 2, weights = ’uniform’, algorithm = ’auto’	Default
Left-segment	SVM	C = 3.5, gamma = 4.0, kernel = ’rbf’	Tuned
Right-segment	LR	C = 1.0, penalty = ’l2’	Default
Arm-side	kNN	n_neighbors = 5, p = 2, weights = ’uniform’, algorithm = ’auto’	Default
Hand-side	DT	criterion = ’gini’, max_depth = None, min_samples_leaf = 1, min_samples_split = 2, splitter = ’best’	Default
Thigh-side	SVM	C=0.5, gamma = 2.0, kernel = ’sigmoid’	Tuned
